# Scientific diasporas in global biodiversity governance: brain circulation, linkage, and knowledge brokerage in Colombia

**DOI:** 10.3389/frma.2026.1844103

**Published:** 2026-07-03

**Authors:** Julian Prieto, Natalia Matiz-Rubio, Camila González, Viviana M. Posada-Pérez, Laura Catalina Quintero Uribe, Miguel Fernández-Niño, Esteban Charria-Girón, Mónica Medina, Ramón Fernando Colmenares-Quintero, Melina Flórez-Cuadros, Yudy Tibaduiza

**Affiliations:** 1 University of Toronto, Toronto, ON, Canada; 2 Red Colombiana de Científicos en el Exterior, Toronto, ON, Canada; 3 Colombian-German Network for Science and Innovation BioGeCo, Stuttgart, Germany; 4 Department of Agricultural and Biological Engineering, The Pennsylvania State University, University Park, PA, United States; 5 Pontificia Universidad Javeriana - Cali, Cali, Colombia; 6 Research Group of Biodiversity Conservation, German Centre for Integrative Biodiversity Research (iDiv), Halle-Jena-Leipzig, Leipzig, Germany; 7 Department of Zoology, Institute of Biology, Martin Luther Universität Halle-Wittenberg, Halle, Germany; 8 Instituto de Agroquimica y Tecnologia de Alimentos, Paterna, Spain; 9 Wageningen University & Research, Wageningen, Netherlands; 10 University of California, Los Angeles, Los Angeles, CA, United States; 11 Universidad Cooperativa de Colombia, Bogotá, Colombia; 12 Global Young Academy, Halle, Germany; 13 Red Académica Científica de Colombia en México, Ciudad de Mexico, Mexico; 14 Secretaria de Ciencia Humanidades Tecnologia e Innovacion, Mexico City, Mexico; 15 Sistema Nacional de Investigadores, Mexico City, Mexico

**Keywords:** biodiversity governance, brain circulation and linkage, capacity building, knowledge brokerage, scientific cooperation, scientific diasporas

## Abstract

The increasing reliance on international scientific collaboration to address biodiversity loss has brought renewed attention to the role of scientific diasporas in global environmental governance. Yet, empirical evidence on how diaspora-mediated practices operate in biodiversity-rich contexts remains limited. This study examines the role of the Colombian scientific diaspora in biodiversity governance by linking empirical patterns to the concepts of brain circulation, brain linkage, and knowledge brokerage. It adopts an exploratory mixed-methods approach that integrates survey (*n* = 62) and qualitative data generated in the context of COP16. The analysis maps diaspora engagement across multiple dimensions, including where collaboration occurs, through which institutional actors it is mediated, how it is enacted, and under what conditions it unfolds. Findings show that diaspora scientists contribute across all 23 targets of the Kunming–Montreal Global Biodiversity Framework, with a strong concentration in capacity building, technology transfer, and international collaboration. Engagement is geographically concentrated in major research hubs but extends to peripheral regions, suggesting a pattern of both concentration and reach. Institutionally, collaborations are primarily mediated through higher education institutions and civil society organizations, with limited direct interaction with national government actors. Building on these findings, the study develops an exploratory typology of diaspora collaboration mechanisms, including academic collaboration, capacity building and technology transfer, co-production with society, material exchange, policy advice, and non-institutional collaboration, offering a structured framework for future studies of diaspora engagement in biodiversity governance.

## Introduction

The accelerating loss of biodiversity has placed international scientific cooperation at the center of global environmental governance. Under the Convention on Biological Diversity (CBD), Parties committed to advancing technical and scientific cooperation (Article 18), facilitating access to and transfer of relevant technologies (Article 16), and strengthening national scientific capacity through exchange of information (Article 17). These commitments were reaffirmed and expanded in the Kunming–Montreal Global Biodiversity Framework (KMGBF), which identified capacity building, technology transfer, and international collaboration as enabling conditions for achieving its 2,030 targets. In this architecture, scientific cooperation is not auxiliary, it is instrumental in its implementation.

Biodiversity research is structurally global and collaborative, given the transboundary nature of ecosystems and conservation challenges. According to Elsevier's 2024 Biodiversity Research Report presented at COP16 in Cali, Colombia, 36% of biodiversity publications worldwide involve authors from more than one country—well above the cross-disciplinary average—and approximately 10% are cited in policy documents, underscoring the field's governance relevance (Barr et al., 2024). This internationalization coexists with persistent asymmetries in knowledge production: Europe, the United States, and China account for a substantial share of global output. Biodiversity-rich regions in the Global South demonstrate high relative engagement but remain embedded in international collaboration networks. Bibliometric analyses of biodiversity research in Central America, for instance, reveal longstanding dominance by institutions in the United States and Europe, with local research leadership expanding gradually over time (Morales-Marroquín et al., 2022). Such patterns reflect broader critiques of scientific extractivism and colonial continuities in biodiversity knowledge production (Büscher and Fletcher, 2015; De Vos and Schwartz, 2022; Miller et al., 2023; Valdez et al., 2024), whereby biodiversity-rich regions contribute field access and biological resources while advanced analytical infrastructure and high-impact publication venues remain concentrated elsewhere (Amano and Sutherland, 2013; Campos-Arceiz et al., 2018; Scholz et al., 2022). However, inequalities in funding, infrastructure, publication visibility, and participation continue to shape whose knowledge is recognized, mobilized, and translated into global scientific and policy processes (Asatani et al., 2026). International collaboration, therefore, operates within an uneven global production of scientific knowledge, and identifying actors capable of mediating, redistributing, or transforming these dynamics becomes critical.

Scientific diasporas represent an underexamined but potentially strategic set of actors. By maintaining active engagement with their countries of origin, diaspora researchers may facilitate brain circulation and linkage while also acting as knowledge brokers across national and sectoral boundaries (Echeverría-King et al., 2022). Diaspora members may contribute to research co-production, mentoring, infrastructure access, funding mobilization, and policy engagement (Bonilla et al., 2022; Gómez-Flores et al., 2022; Tejada et al., 2013), activities directly aligned with the CBD's cooperation and capacity-building mandates. These efforts are particularly valuable for bridging scientific communities and expanding the reach of biodiversity research from underrepresented regions. In this way, scientific diasporas help address systemic barriers and amplify local and Indigenous perspectives that are often marginalized in global discussions, thereby strengthening the operationalization of the KMGBF. The distinctive role of the scientific diaspora may provide a vital mechanism for connecting local realities to international frameworks and advancing inclusive, impactful biodiversity science.

Empirical evidence on how diaspora-mediated practices are embedded in biodiversity governance contexts remains limited, particularly in biodiversity-rich countries (Morales-Marroquín et al., 2022). This study adopts a mixed-methods approach, using the case of Colombia to explore, map, and conceptualize diaspora practices within international biodiversity governance. The analysis combines survey data, which maps diaspora scientists' contributions to KMGBF-related processes, with qualitative evidence that provides deeper insight into how these contributions are enacted in practice. Building on this empirical foundation, we develop an exploratory typology that captures key mechanisms of engagement and provides a structured lens to examine where diaspora collaboration occurs, through which institutional actors it is channeled, how it is enacted, and under what conditions it unfolds.

## Brain circulation, brain linkage, and knowledge brokerage

Brain circulation challenges traditional “brain drain” narratives by emphasizing the multidirectional movement of scientists and the possibility of knowledge exchange through return, temporary mobility, and sustained professional collaboration (Meyer, 2001). Rather than framing migration exclusively as a loss of human capital for developing countries, this perspective conceptualizes scientific mobility as a potential developmental resource embedded in transnational research networks. Closely related, the concept of brain linkage shifts attention from physical return to sustained engagement across borders, highlighting how diaspora scientists may contribute to their countries of origin through joint research, mentoring, co-supervision, infrastructure access, and institutional partnerships without permanently relocating.

Complementing these practices, knowledge brokerage refers to the role of actors who operate across institutional and governance boundaries to facilitate the translation, coordination, and circulation of expertise between scientific and policy domains (Gluckman et al., 2021). In biodiversity governance, this function plays an important role given the need to translate global biodiversity targets into national implementation strategies, monitoring systems, and regulatory frameworks. Diaspora scientists, embedded simultaneously in international research systems and domestic institutional contexts, may therefore occupy strategic intermediary positions within these processes.

Taken together, brain circulation, brain linkage, and knowledge brokerage provide a conceptual foundation for analyzing how diaspora scientists engage in biodiversity governance not simply as participants in transnational collaboration networks, but as actors operating within—and potentially mediating—asymmetrical global knowledge systems. This perspective is particularly relevant in biodiversity research, where international collaboration is both structurally necessary and shaped by persistent inequalities in scientific capacity and access to global research infrastructures.

## Methods and data

This research adopts an exploratory mixed-methods design (Creswell, 2015) and presents a case study of Colombia aimed at examining the role of scientific diasporas in biodiversity governance by mapping and analyzing the mechanisms and institutional dynamics shaping their collaborations with institutions in Colombia. The empirical material was generated in the context of the Sixteenth Conference of the Parties to the Convention on Biological Diversity (COP16), held in Cali, Colombia, in 2024, which created a moment of mobilization for the Colombian scientific diaspora. In preparation for the conference, several diaspora organizations, both established and in the process of formalization, launched initiatives aimed at documenting the contributions of Colombian scientists living abroad to biodiversity research, international scientific collaboration, and science–policy processes. Among these, two organizations, Red Colombiana de Científicos en el Exterior and the Red Colombo-Alemana de Ciencia e Innovación BioGeCo (hereafter “the BioGeCo Network”), developed complementary data collection efforts through surveys, focus groups, and public engagement events. Although these initiatives were conducted independently, their thematic convergence enabled the integration of the resulting datasets. We therefore combine survey and qualitative data following an *a posteriori* comparative approach that integrates independently generated data to enhance analytical insight (Montero and Baiocchi, 2022).

### Survey data

Two independent surveys were conducted between July and September 2024 and distributed through diaspora networks and partner organizations. The sample was obtained through non-probabilistic sampling based on voluntary participation within both diaspora networks. One survey targeted members of the Red Colombiana de Científicos en el Exterior, while the second focused on the BioGeCo Network in Germany. The combined sample includes 62 respondents (42 global, 20 Germany-based). Both datasets were harmonized into a single analytical dataset by aligning shared variables on KMGBF contributions, type of institutional engagement, and the regional scope of the collaborations.

### Focus groups and public engagement events

Qualitative data were generated through two diaspora engagement initiatives conducted in preparation for COP16: a virtual Pre-COP focus group organized by the Red Colombiana de Científicos en el Exterior and an in-person event organized by BioGeCo Network in Germany. The virtual focus group brought together 30 of the 42 survey respondents and included structured discussions on best practices, challenges, and policy recommendations related to diaspora collaboration in biodiversity governance. The in-person event, open to the public, featured seven presentations by diaspora researchers reflecting on their collaboration experiences and policy insights, with approximately 20 participants attending the session. All qualitative materials were transcribed, systematized, and analyzed using a shared coding framework organized around three analytical categories: best practices, main challenges, and policy recommendations.

### Data analysis

To examine patterns of diaspora engagement in biodiversity governance, quantitative and qualitative data sources were integrated through an iterative mixed-methods strategy. Survey data were used to identify patterns of collaboration, institutional engagement, and territorial distribution, while qualitative data generated through focus groups and public engagement activities provided deeper insight into the practices, enabling conditions, challenges, and institutional dynamics shaping diaspora collaboration. Rather than treating both datasets independently, the analysis relied on iterative triangulation in which quantitative patterns informed qualitative interpretation, while qualitative evidence helped to contextualize and refine recurring patterns identified in the survey data. This integrative process informed the development of a study-defined typology of diaspora-mediated collaboration mechanisms.

The quantitative analysis combined complementary visualization and comparative approaches to examine how diaspora collaborations relate to KMGBF targets, Colombian territorial contexts, institutional actors, and collaboration practices. Through iterative triangulation across these variables, the analysis examined how reported contributions to particular biodiversity governance priorities were connected to collaborations across different Colombian departments. Institutional categories associated with each territorial reference were then grouped to examine the types of organizations most frequently involved in diaspora engagement across regions. To further contextualize these territorial patterns, departmental science, technology, and innovation investment data were paired with engagement metrics derived from survey responses to explore whether the concentration of reported collaborations reflected broader national investment distributions. This comparative analysis enabled the study to examine how existing scientific infrastructure and institutional capacity may shape the territorial distribution of diaspora collaborations.

Qualitative materials were manually coded according to recurring practices, enabling conditions, challenges, and policy recommendations associated with diaspora engagement. Through iterative comparison across survey and qualitative data sources, these coded patterns were grouped into broader categories of collaboration mechanisms, including academic collaboration, capacity building and technology transfer, co-production with society, policy advice, material request, and non-institutional collaboration. Coding was conducted collaboratively by the authors using a shared analytical framework, with iterative discussions used to refine categories and ensure consistency across interpretations and data sources. This integrated analytical strategy enabled the study to move beyond descriptive mapping toward a more differentiated understanding of how diaspora-mediated collaboration operates across institutional, territorial, and governance contexts.

### Positionality

All authors of this study are, or have been, part of the Colombian scientific diaspora. Several authors have also taken active leadership roles in articulating collective diaspora efforts, including within organizations such as Red Colombiana de Científicos en el Exterior, the BioGeCo Network, Red Académica y Científica de Colombia en México, and Red BERSTIC. This positionality facilitated access to diaspora networks and enabled the co-production of empirical data in the context of COP16-related initiatives. At the same time, the authors' embeddedness in these organizations is acknowledged as introducing a potential risk of subjectivity in the interpretation of the data. To mitigate this, the analysis was conducted with attention to methodological rigor and reflexivity, recognizing the dual role of the authors as both participants in and analysts of diaspora engagement processes.

## Results

Drawing on the integration of survey and qualitative data, this section identifies and characterizes observed mechanisms through which diaspora scientists engage in biodiversity research and governance, while examining how these mechanisms operate within an uneven global knowledge production system.

### Contributions to KMGBF targets and geographical connectivity

**Figure 1:**
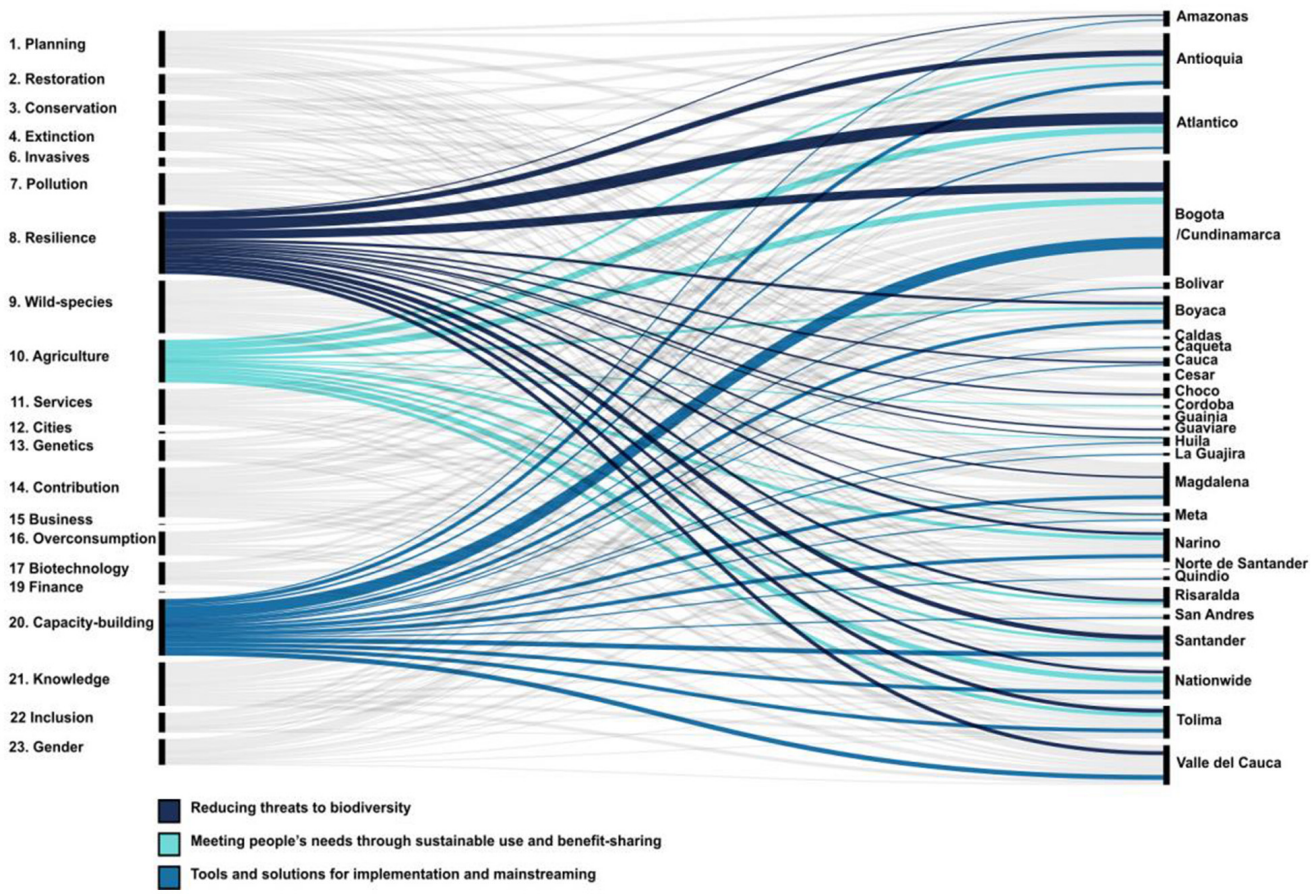
Technical structure of the alluvial mapping between KMGBF targets and Colombian departments. The diagram illustrates the categorical flow from the 23 targets **(left)** to geographical nodes **(right)**. Ribbon width is proportional to the frequency of reported connections, derived from the collaborations and interpretations of 62 survey respondents. Stream colors highlight the most frequent targets within the three KMGBF strategic pillars.

Figure 1 maps how diaspora researchers reported that their research contributes to the KMGBF targets through collaborations with institutions in Colombia. The analysis is organized around the three strategic pillars of the KMGBF: reducing threats to biodiversity (targets 1–8), meeting people's needs through sustainable use and benefit-sharing (targets 9–13), and tools and solutions for implementation and mainstreaming (targets 14–23). Across these pillars, the results reveal differentiated but interconnected patterns of diaspora engagement distributed across biodiversity governance priorities and Colombian departments.

Within the pillar on reducing threats to biodiversity, Target 8 (resilience) concentrates a substantial share of reported connections, particularly in Bogotá–Cundinamarca, Antioquia, and Atlántico. This suggests that resilience-related research and collaborations are closely associated with the country's main research and policy hubs. At the same time, connections linked to Target 8 were also reported in ecologically sensitive and peripheral regions such as Amazonas, Chocó, and La Guajira.

The pillar on meeting people's needs through sustainable use is structured primarily around Target 10 (agriculture), with collaborations more closely associated with productive landscapes and sector-specific activities related to agriculture, forestry, and natural resource management. Departments such as Atlántico, Santander, Córdoba, and Cauca emerge as important nodes within these collaboration patterns, suggesting that diaspora engagement in this domain operates through territorially grounded development and resource-use dynamics.

The broadest geographical reach is observed within the pillar on tools and solutions for implementation, particularly through Target 20 (capacity-building). Collaborations associated with this target extend across nearly all Colombian departments, including a nationwide node representing respondents engaged through institutions operating across multiple regions. Consistent linkages with Target 13 on benefit-sharing from genetic resources and Target 14 on integrating biodiversity into decision-making further indicate that these collaborations extend beyond technical research activities into domains associated with governance coordination.

From a broader geographical perspective, respondents reported contributions spanning all KMGBF targets and collaborations involving all Colombian departments, indicating a wide territorial and thematic distribution of diaspora engagement. Beyond this, the results reveal a simultaneous pattern of concentration and reach. While reported collaborations remain strongly clustered around Bogotá–Cundinamarca, Antioquia, and Valle del Cauca, thinner but geographically widespread connections extend to regions such as Nariño, Putumayo, Guaviare, and San Andrés and Providencia.

### Institutional mediation of diaspora collaboration

**Figure 2:**
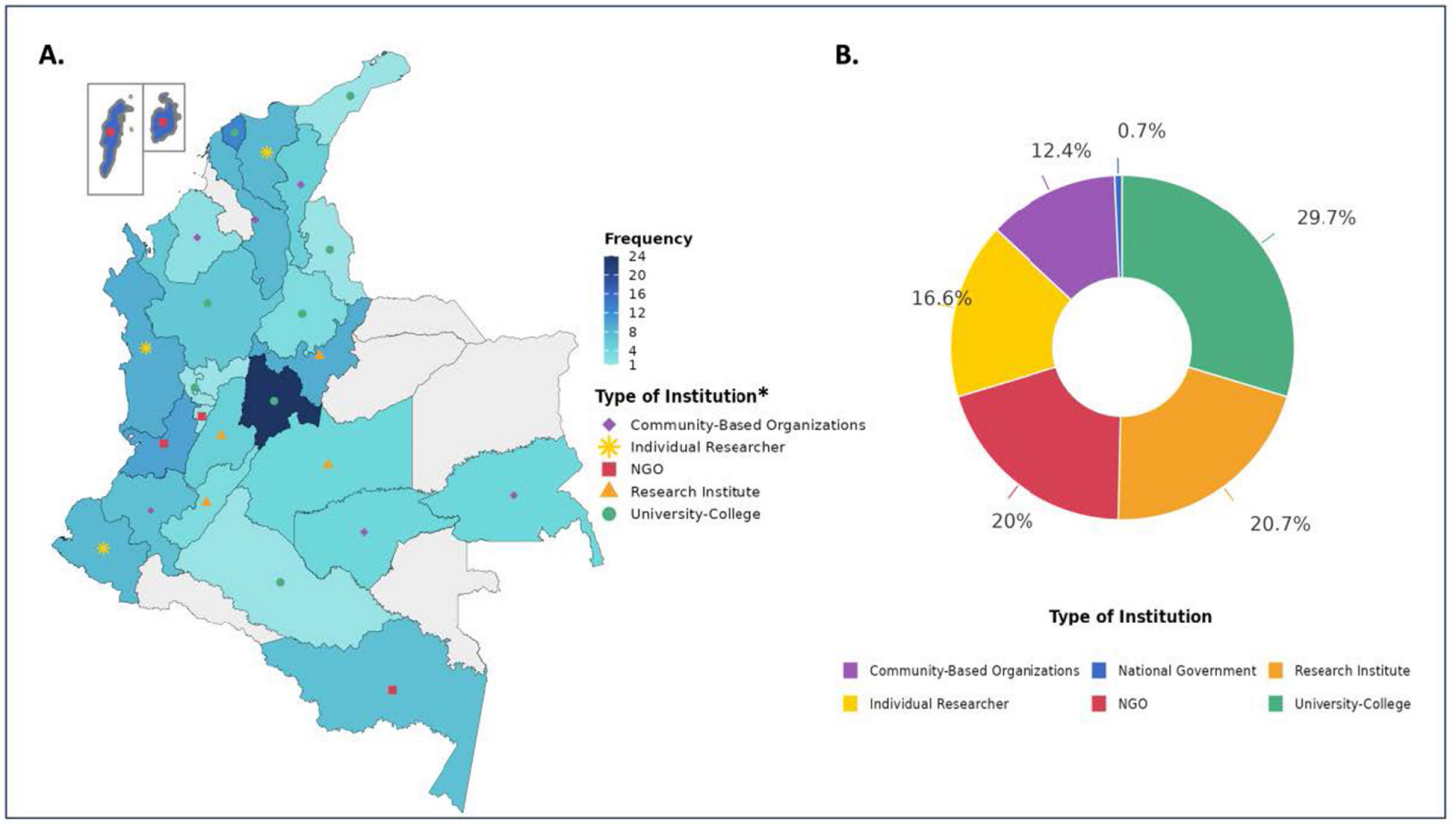
Geographical distribution of departments most frequently collaborating with diaspora researchers and predominant institution types. **(A)** Heatmap indicating frequency of departmental collaborations as reported by 62 survey respondents, whose responses could include collaborations across multiple departments. Icons denote the most frequently reported institution type for each department. **(B)** Donut chart summarizing the relative frequencies of institution types reported in the survey.

Figure 2 reveals the institutional configurations through which regional collaborations are mediated. Rather than simply indicating where collaborations occur, the figure highlights how they are structured by means of different types of organizations, providing insight into the institutional infrastructures that enable diaspora engagement.

Given their shared academic orientation, universities and research institutes emerge as dense nodes that attract and concentrate above fifty percent of diaspora collaboration. In these contexts, collaboration is primarily structured through formal institutional partnerships based on co-authorship, joint research, and academic coordination. Collaborations with these institutions respond to two geographical dynamics. First, these collaborations are concentrated in departments that have a robust network of academic institutions and a consolidated research ecosystem, such as Bogotá–Cundinamarca, Antioquia, and Atlántico. Second, collaborations also extend to more peripheral regions such as La Guajira, Norte de Santander, and Caquetá, where their prominence reflects the presence of a limited number of organizations acting as primary interfaces for collaboration. Although not institutionally mediated, collaborations with individual researchers (16.6%) follow similar peer-based collaboration dynamics.

Alongside this, a substantial share of collaborations is channeled through civil society actors, with NGOs (20.0%) and community-based organizations (12.4%) representing almost one-third of reported engagements. This highlights the importance of non-academic intermediaries in facilitating diaspora collaboration, particularly in contexts where formal research institutions are less prominent or accessible. The geographical distribution where these organizations serve as primary mediators includes Amazonas, Guainía, Cauca, Córdoba, Bolívar, and Cesar. These regions do not conform to a single territorial profile: some are geographically remote and characterized by limited state and research infrastructure, while others have a stronger institutional capacity but are marked by strong traditions of community organization and civil society engagement. In contrast, collaboration with national government institutions remains minimal (0.7%), indicating a limited direct interface between diaspora scientists and central state structures.

**Figure 3:**
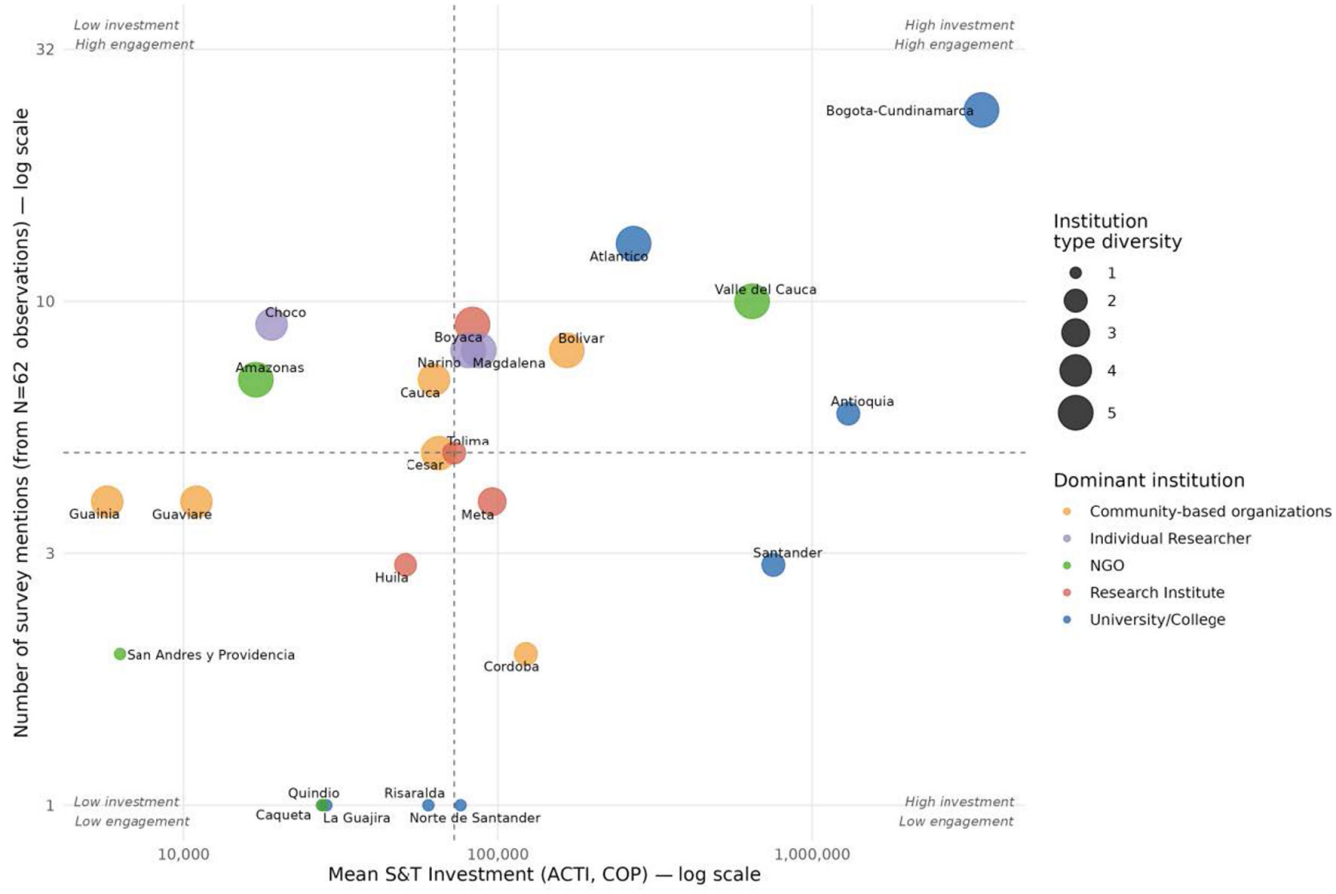
Median-split bivariate analysis of departmental Science and Technology (S&T) investment and number of survey's mentions across Colombian departments. The figure is partitioned into four quadrants defined by median values of both axis (dashed lines), according to departmental investment-mention profiles. Both axes are displayed on a logarithmic scale to account for the skewed distribution of values. The x-axis represents mean S&T investment per department (OCyT, 2024); while the y-axis captures the number of survey's mentions per department derived from the scientific diaspora survey.

To further contextualize the observed collaboration patterns, a median-split bivariate analysis was conducted comparing departmental S and T investment with the frequency of survey mentions associated with diaspora engagement (Figure 3). This figure reveals a differentiated territorial relationship between departmental scientific investment and reported diaspora engagement. Departments such as Bogotá–Cundinamarca, Atlántico, Valle del Cauca, and Antioquia combine relatively high levels of science and technology investment with high frequencies of survey mentions, consistent with their role as consolidated scientific and institutional hubs within the national research system.

Additionally, diaspora engagement was not exclusively concentrated in highly invested regions. Departments such as Chocó, Amazonas, and Cesar exhibited comparatively high levels of survey visibility despite lower levels of science and technology investment. In several of these regions, collaborations were more frequently through NGOs and community-based organizations rather than through dense academic infrastructures, reinforcing the importance of civil society actors as intermediary institutional nodes in territorially peripheral or underinvested contexts.

### Typology of diaspora collaboration mechanisms

**Figure 4:**
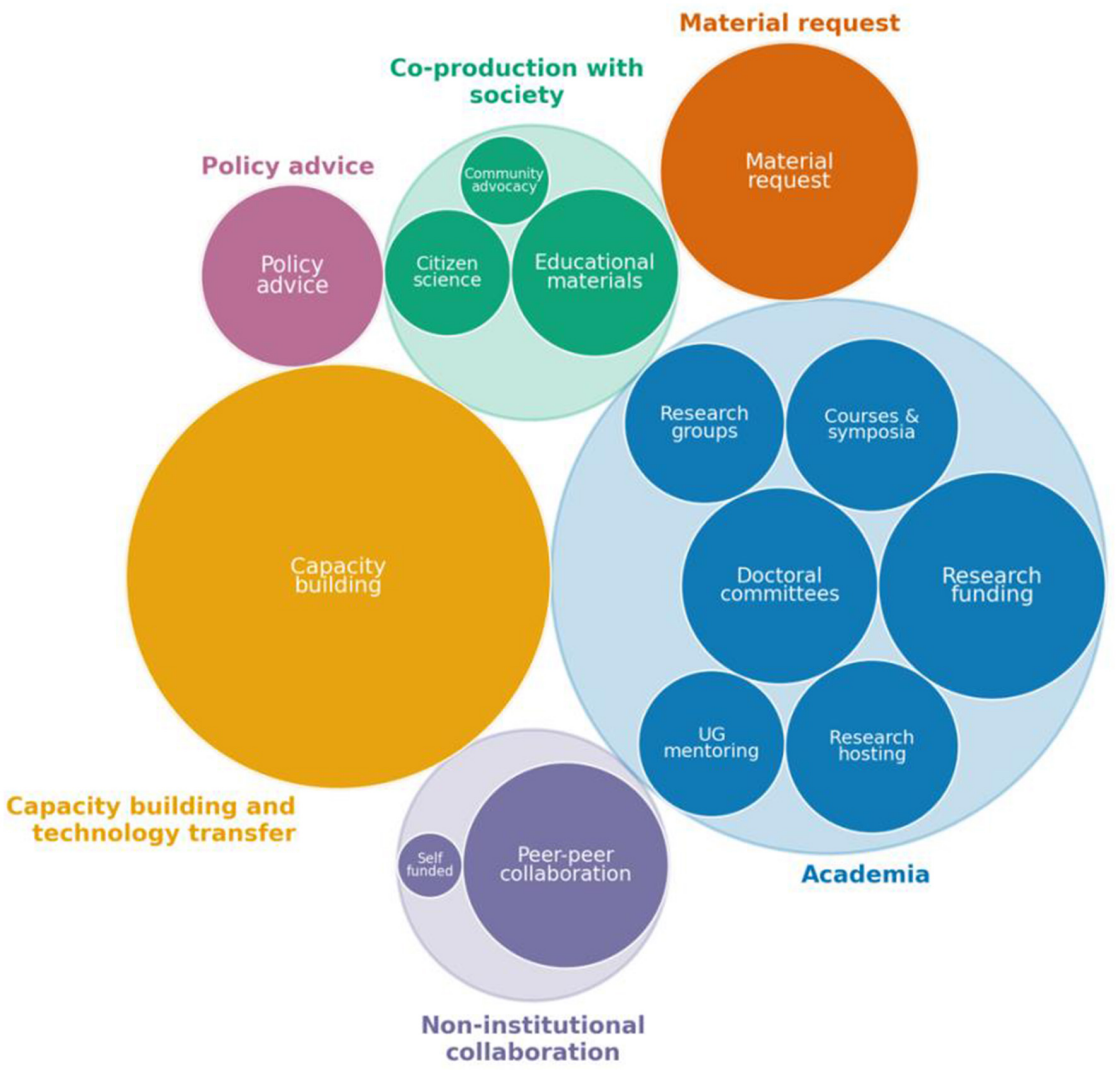
Collaboration mechanisms between members of the Colombian scientific diaspora and actors in Colombia. Circle size represents the relative frequency of each mechanism identified in the analysis. Shaded clusters indicate broader domains of engagement based on categories developed in this study, including academic collaboration, capacity building and technology transfer, co-production with society, policy advice, material request, and non-institutional collaboration.

The distribution of collaboration mechanisms presented in Figure 4 provides insight into how members of the Colombian scientific diaspora engage with actors in Colombia, moving from institutional structures to the concrete practices through which collaboration is enacted. Based on 112 recorded instances, we identify a set of recurring mechanisms that can be grouped into a study-defined typology of diaspora engagement, derived from the combined interpretation of survey data and qualitative evidence.

Academic collaboration appears as the most prominent domain in the sample, suggesting the continued centrality of formal institutional partnerships in structuring diaspora engagement. Mechanisms such as joint research funding, participation in research groups, doctoral co-supervision, and short-term research stays indicate that diaspora contributions remain strongly anchored in established academic infrastructures. At the same time, the presence of non-institutional collaboration, primarily peer-to-peer interactions between individual researchers, highlights a complementary layer of engagement that operates outside formal organizational frameworks.

Capacity building and technology transfer appear as the second major type of engagement, highlighting the role of diaspora actors not only in knowledge production but also in strengthening local research and innovation capacities. These mechanisms often combine elements of training, mentorship, and applied knowledge transfer, where knowledge and skills are mobilized across contexts and adapted to local needs.

A further dimension of engagement is represented by co-production with society, which includes participatory research practices such as citizen science, community advocacy, and the development of locally relevant educational materials. These mechanisms reflect a shift toward more socially embedded forms of knowledge production, where diaspora actors mediate between scientific expertise and local actors, institutions, and territories.

In contrast, material exchange and policy advisory roles occur less frequently, indicating more specialized or context-dependent forms of engagement. Material exchange refers to the transfer, loan, or request of physical samples, such as biological specimens, microbial strains, soil, water, and derived data, which are typically governed by permits, material transfer agreements, and documentation of provenance. These mechanisms are often shaped by regulatory and logistical constraints, while policy advice requires access to institutional decision-making spaces.

### Practices, challenges, and enabling conditions of diaspora collaboration

Based on the qualitative material analyzed, our findings provide insight into how the collaboration mechanisms identified in the previous section operate in practice, offering a grounded perspective on the conditions that enable and constrain diaspora engagement. During the coding process, patterns of good practices, challenges, and enabling conditions were found to align with the typology of collaboration mechanisms developed from the survey data. Qualitative insights are presented in Table 1, systematically organized according to these categories, allowing for an integrated analysis of how academic collaboration, capacity building, and technology transfer, co-production with society, policy advice, material request, and non-institutional collaboration are enacted in practice under uneven institutional and infrastructural conditions. In doing so, we observed that diaspora collaboration appears differentiated not only by form but also by the contexts in which it unfolds, where access to infrastructure, funding, regulatory frameworks, and institutional recognition plays an important role.

**Table 1:** Operational dynamics of diaspora collaboration mechanisms.

Collaboration mechanism	Good practices	Examples	Outcomes of good practices	Challenge
Academic collaboration	Active and continuous engagement with Colombian universities	Engaging with research groups in Colombia; maintaining ties with one's Alma Mater with framework agreements; participating in doctoral committees and co-supervising undergraduate research; joint fieldwork.	Strengthens institutional bonds; promotes collaborative efforts over time.	Perceived risks of collaborating with colleagues abroad; scientists abroad are not categorized in CVLAC; international experience is not recognized; binational collaboration depends on third-party funding or individual efforts.
	Development of equitable and contextualized joint research projects	Designing mutually beneficial research projects; establishing transparent collaboration parameters, including equitable authorship practices; recognizing partners' different motivations and incentives; identifying relevant public-policy instruments in the research area (Valdez et al., 2024).	Maintains credibility and avoids perceptions of extractive collaboration; leverages resources; generates context-relevant impact.	Differences in work rhythms and timelines; heavier teaching loads in Colombia; differences in incentives and evaluation metrics.
	Co-financing of research projects with international resources	Combining Colombian and foreign institutional resources; leveraging binational programs.	Increases project feasibility; reduces asymmetries in collaborations.	Lack of mechanisms to access local calls; no mechanisms to serve as MinCiencias evaluators.
	Raising awareness of systemic barriers	Informing peers abroad about structural challenges and inequities in collaboration frameworks.	Legitimizes discussions on inequity; shifts community norms toward more equitable collaboration practices.	Researchers lack practical frameworks or institutional mandates to change collaboration practices.
Non-institutional collaboration				
	Active and continuous engagement with peers in Colombia	Developing peer-to-peer collaborations; maintaining ties with one's Alma Mater with framework agreements; maintaining regular virtual communication and periodic in-person visits.	Builds trust with researchers.	Lack of connectivity with regional researchers.
Capacity building and technology transfer	Development of open-source tools, data standardization, decentralized infrastructures	Converting fragmented, institution-specific data into interoperable formats; releasing opensource code, documented APIs, and shared cloud-based processing; establishing federated hosting (regional nodes) (Suarez et al., 2024).	Democratizes access to data and tools; lowers technical barriers for resource-constrained partners; reduces reliance on a single, costly central repository.	Lack of standardized biological ontologies linking physical specimens, Digital Sequence Information (DSI), and downstream applications.
	Reciprocity	Investing in local infrastructure for storage, sequencing, and bioinformatics; co-financing biobanks; returning data and results to local institutions; involving local researchers across the value chain.	Enhances national sovereignty over genetic resources; ensures fairness; increases long-term impact.	Samples and data come from biodiverse-rich countries, typically in the Global South; analysis, publications, and benefits still concentrate in institutions usually located in the Global North.
	Knowledge brokerage	Creating structured roles and platforms beyond simple data-sharing; training workshops for tool users.	Supports equitable participation and multi-directional knowledge flow.	Colombia lags global standards in biodiversity monitoring technologies and infrastructure.
Policy Advice		Framing biodiversity as a strategic asset for the bioeconomy in policy dialogues.	Helps policymakers recognize biodiversity as a driver of scientific and economic development.	No clear roadmap for responsible bioprospecting that integrates conservation, innovation, and industrial development objectives.
		Using high-visibility platforms, such as international conferences to advocate for inclusive and equitable collaboration standards.	Catalyzes standard-setting dialogues that shift community norms.	Financial, visa, language, and logistical barriers limit participation of underrepresented researchers in conferences and workshops.
	Identifying relevant public-policy instruments	Mapping policy tools, such as national biodiversity strategies; using policy instruments to secure resources or institutional support.	Strengthens alignment between research and national development goals; increases policy uptake of scientific results.	Absence of a long-term Science, Technology and Innovation policy; overlapping mandates and inconsistent guidance from different Ministries.
Co-production with society	Co-creation with stakeholders outside academia	Designing, governing, and hosting data infrastructures with local communities and national agencies; applying technologies such as camera traps, phenocams, bioacoustics and, eDNA.	Enhances community empowerment and monitoring capacity; enables scientists to obtain data more efficiently; improves adoption and long-term use of methodologies and tools.	Lack of standardized indicators for global biodiversity monitoring
	Linking scientific research with traditional knowledge	Systematic exploration of beneficial fungi from medicinal plants in tropical rainforests in Colombia; collaboration with local knowledge holders to guide sampling and prioritization (Charria-Girón et al., 2023).	Accelerates discovery; highlights the value of commercially unexplored biological resources; strengthens shared ownership of research outputs.	Fungi are not formally recognized as a separate kingdom, creating legal uncertainty.
Material request	Formal partnerships with local institutions	Institutional framework agreements with Colombian institutions and agencies for managing collection permits.	Facilitates access to and improves management of collection permits; enables sustained collaborative efforts.	Divergent national interpretations of the Nagoya Protocol leads to different requirements and compliance expectations
	Early alignment with Access and Benefit-Sharing (ABS) and legal frameworks	Identifying applicable ABS and Nagoya Protocol requirements at project inception; establishing Material Transfer Agreements (MTA) before collection or shipment; defining scope of use, ownership, benefit-sharing, and intellectual property; addressing DSI.	Reduces legal uncertainty; prevents conflicts; strengthens long-term collaboration.	Regulatory and bureaucratic complexity; permitting processes are often lengthy, paper-based, and insufficiently digitalized; institutional fragmentation across ministries, typically environment, agriculture, science, and health.

Across collaboration mechanisms, several enabling practices emerge consistently, highlighting the importance of sustained engagement, reciprocity, and contextual sensitivity in shaping effective diaspora partnerships. In academic and non-institutional collaborations, long-term relationships—often anchored in prior institutional affiliations or peer networks—appear to play an important role in building trust and facilitating coordination over time, reflecting dynamics associated with both brain linkage and circulation. Similarly, in capacity building and technology transfer, respondents emphasized practices such as co-financing, open-source tool development, investment in local infrastructure, and interoperable data systems. These practices were often described as mechanisms for reducing technical and infrastructural asymmetries. In co-production with society, effective collaboration tends to be characterized by the active involvement of local communities and stakeholders throughout the research process, aligning closely with knowledge brokerage processes that connect scientific expertise with territorial needs and local governance contexts. Together, these practices suggest that diaspora engagement often depends not only on access to resources, but also on relational and institutional alignment across contexts.

While these practices highlight enabling conditions, they are simultaneously shaped by structural constraints that limit their scope and sustainability. Across mechanisms, respondents pointed to asymmetries in funding, infrastructure, and institutional recognition as key barriers. For instance, differences in evaluation systems and the limited recognition of international experience within national research frameworks create disincentives for sustained collaboration. In the domains of capacity building and material exchange, respondents emphasized persistent infrastructural and regulatory challenges, including fragmented biodiversity data systems, the absence of standardized ontologies linking physical specimens and Digital Sequence Information (DSI), and administrative complexity associated with Access and Benefit-Sharing (ABS) frameworks and the Nagoya Protocol. Similarly, respondents highlighted that permitting systems for the collection and transfer of biological samples often remain bureaucratically fragmented, paper-based, and difficult to navigate across ministries and regulatory agencies. These constraints were described as particularly challenging for international collaborations involving biological samples, genetic resources, and transnational research coordination.

The tensions surrounding diaspora engagement also become particularly visible in the domain of policy advice. Respondents described how diaspora scientists may leverage international conferences and transnational scientific networks to advocate for more inclusive collaboration standards and biodiversity governance agendas. Together, this finding aligns with the survey data, where collaborations with central and regional government institutions appeared as the least frequently reported form of institutional engagement. Instead, many of the knowledge brokerage functions identified in the qualitative material appear to operate through collaborations with universities, NGOs, and community-based organizations, which frequently serve as intermediary actors connecting scientific expertise with local governance and implementation processes. Respondents nevertheless emphasized that fragmented institutional mandates, the absence of long-term STI strategies, and financial, linguistic, and visa-related barriers continue to limit more direct participation in formal policymaking and international scientific governance spaces.

In addition to identifying constraints, the qualitative data also point to a set of policy-oriented recommendations aimed at strengthening the institutional conditions that enable diaspora collaboration. Many of these recommendations are directed toward the Ministry of Environment and focus on improving regulatory frameworks governing access to biodiversity resources and data derived from them. Participants emphasized the need to simplify and digitalize permitting processes for the collection and transfer of biological samples, including the creation of mechanisms tailored to the scientific diaspora. Additional proposals include strengthening national infrastructure for the preservation of biological collections, particularly through certified biobanks operating under internationally recognized standards, and implementing digital traceability systems linking physical samples to associated DSI. Participants also highlighted the importance of ensuring the inclusion of Indigenous peoples and local communities in the governance of genetic resources through mechanisms that support equitable participation and benefit-sharing.

Complementary recommendations target the Ministry of Science, Technology, and Innovation (STI) and emphasize the need for long-term, system-level interventions. Participants highlighted the importance of establishing stable funding mechanisms, developing coherent STI strategies that transcend short-term political cycles, and creating structured pathways for diaspora engagement within the national research system, including enabling diaspora scientists to contribute as evaluators and technical advisors in regulatory and ABS-related processes. Across both policy domains, these recommendations indicate that strengthening diaspora collaboration will likely require coordinated action to address regulatory, infrastructural, and institutional gaps, enabling more sustained, equitable, and impactful forms of engagement. The full list of enabling conditions gathered in this study for diaspora collaboration, including their potential effects on fostering collaboration mechanisms, is provided in Annex 2 for the reference of policymakers and decision-makers.

## Discussion

This study contributes to a more nuanced understanding of the role that scientific diasporas may play in global biodiversity governance by providing an exploratory mixed-methods analysis of diaspora-mediated collaboration in a biodiversity-rich context. Drawing on survey and qualitative data generated through two Colombian diaspora organizations, the study does not seek to provide a statistically representative portrait of the Colombian scientific diaspora. Rather, it offers an empirically grounded characterization of how diaspora engagement is structured across territorial, institutional, and relational dimensions, while identifying some of the enabling conditions and constraints shaping these collaborations in practice. In doing so, the analysis responds to growing calls for more empirical research examining how diaspora-mediated scientific practices operate within uneven global knowledge systems and biodiversity governance contexts (Bonilla et al., 2023; Seguin et al., 2006; Teferra, 2005).

Several consistent patterns emerge from the findings. First, respondents reported contributions spanning all 23 targets of the Kunming–Montreal Global Biodiversity Framework (KMGBF), suggesting a broad alignment between diaspora engagement and contemporary biodiversity governance priorities. Among these, capacity building, technology transfer, and international scientific cooperation emerged as particularly prominent dimensions of engagement. This finding reinforces arguments within the scientific diaspora literature that diaspora contributions often operate less through permanent return than through transnational mechanisms of knowledge circulation, institutional linkage, and collaborative capacity building (Barré et al., 2003; Meyer, 2001; Shin and Moon, 2018; Tejada et al., 2013). In this sense, the findings support conceptualizations of scientific diasporas as actors capable of mobilizing expertise, infrastructure, and international networks across borders without requiring permanent reintegration into national research systems.

At the same time, the geographical distribution of collaborations reveals a dual pattern of concentration and territorial reach. Collaborations remain strongly concentrated around Bogotá–Cundinamarca, Antioquia, and Valle del Cauca, reflecting the historical concentration of scientific infrastructure, funding, and institutional capacity within Colombia's principal research hubs. However, respondents also reported collaborations extending toward peripheral and ecologically significant regions such as Amazonas, Chocó, La Guajira, and Guaviare. Although differentiated by intensity, these patterns suggest that diaspora collaborations may contribute to expanding the circulation of scientific expertise and institutional linkages beyond consolidated urban scientific centers. This observation aligns with broader critiques of uneven biodiversity knowledge production, in which biodiversity-rich territories frequently remain structurally peripheral within global scientific systems despite their ecological importance (Amano and Sutherland, 2013; De Vos and Schwartz, 2022; Miller et al., 2023). Rather than fully overcoming these asymmetries, diaspora engagement appears to operate within, and in some cases partially mediate, the structural inequalities shaping biodiversity research and international scientific collaboration.

The findings further demonstrate that diaspora engagement is strongly facilitated through higher education institutions and civil society organizations, which emerge as key institutional hubs for collaboration. In contrast, direct engagement with national and regional government actors appears limited in comparison. This pattern indicates that many of the knowledge brokerage functions associated with diaspora engagement operate through intermediary organizational networks rather than through formal state structures. Particularly in regions characterized by weaker scientific infrastructure or fragmented governance capacities, NGOs and community-based organizations appear to function as important actors connecting scientific expertise with territorial implementation processes and local governance needs. These findings resonate with broader discussions within the knowledge brokerage literature emphasizing that the circulation and translation of expertise often depend on intermediary organizations capable of operating across fragmented institutional environments (Gluckman et al., 2021; Kuznetsov, 2006).

Importantly, the qualitative findings suggest that diaspora collaboration is shaped not only by opportunities for engagement but also by persistent structural tensions. Respondents consistently identified asymmetries in funding, infrastructure, institutional recognition, and regulatory capacity as important barriers to sustained collaboration. In domains such as material exchange and biodiversity data governance, these tensions become particularly visible through the administrative complexity associated with Access and Benefit-Sharing (ABS) frameworks, permitting systems, and the governance of Digital Sequence Information (DSI). Moreover, respondents highlighted that differences in institutional incentives, evaluation systems, and access to international scientific spaces continue to shape unequal conditions of participation within collaborative research processes. These findings reinforce critiques of celebratory narratives surrounding scientific mobility and international collaboration by illustrating how diaspora engagement remains embedded within broader asymmetries of global knowledge production (Docquier and Rapoport, 2012; Gaillard and Gaillard, 1997).

Beyond its empirical findings, this study contributes to ongoing theoretical discussions surrounding brain circulation, brain linkage, and knowledge brokerage. The proposed typology of diaspora collaboration mechanisms highlights that diaspora engagement extends beyond formal academic collaboration into domains such as capacity building, co-production with society, policy advice, material exchange, and informal peer-to-peer collaboration. Taken together, these findings suggest that scientific diasporas function not only as connectors of knowledge across borders, but also as actors shaping how collaboration is organized and sustained. Rather than simply transferring expertise between countries, diaspora engagement reshapes how collaboration pathways and institutional interfaces are organized. In this sense, the study proposes an exploratory conceptualization of scientific diasporas as infrastructural mediators operating between global governance frameworks, national institutions, and territorially situated collaboration processes.

The study also contributes methodologically by proposing a structured typology of diaspora collaboration mechanisms that may support more systematic and comparable analyses of diaspora engagement across different contexts. By proposing a six-category typology grounded in empirical observation and situated in dialogue with existing classification frameworks (Barré et al., 2003; Katz and Martin, 1997; Seguin et al., 2006), the study offers a replicable analytical tool for future comparative research on diaspora engagement across national and disciplinary contexts.

Several limitations should nevertheless be considered when interpreting these findings. First, the study relies on a non-probabilistic, network-based sample drawn from two diaspora organizations and therefore does not provide statistically representative estimates of the broader Colombian scientific diaspora. The findings should instead be understood as indicative of engagement patterns among actively connected members within these organizational networks. Second, the geographical concentration of respondents in Germany introduces additional contextual bias associated with the specific characteristics of German scientific cooperation frameworks and bilateral research programs. Third, both survey and qualitative materials rely on self-reported experiences, which may privilege more visible or successful forms of collaboration. Finally, as acknowledged in the positionality section, all authors are members of the Colombian scientific diaspora, providing valuable insider perspectives while also introducing potential interpretive biases. Future research would benefit from larger comparative samples, longitudinal approaches, and the incorporation of perspectives from institutional and community-based collaborators within Colombia itself.

## Conclusions

This study advances understanding of the role of scientific diasporas in global biodiversity governance by providing an empirically grounded and analytically structured account of how diaspora collaboration is organized and enacted. Adopting an exploratory mixed-methods design appropriate to an understudied phenomenon, the analysis identifies key dimensions of diaspora engagement. These include where collaboration occurs, through which institutional actors it is mediated, how it is enacted, and under what conditions it unfolds. Building on these findings, the study proposes a typology of collaboration mechanisms that captures the diversity of these practices.

Three principal contributions emerge from this analysis. First, the study demonstrates that diaspora engagement in biodiversity governance is multidimensional and functionally diverse, encompassing not only conventional academic partnerships but also capacity building, participatory co-production, material exchange, and, to a more limited extent, policy advice.

Second, the analysis reveals that the institutional architecture of diaspora collaboration is shaped by territorial inequalities and governance gaps. The predominance of higher education and research institutions and civil society organizations as mediators of engagement, coupled with the near-absence of direct interaction with national government actors, suggests that diaspora-mediated knowledge brokerage operates through indirect channels, a finding with important implications for science policy. If policymakers seek to harness diaspora expertise for biodiversity governance, they must create structured pathways for engagement within formal governance architectures, rather than relying on the informal and often precarious channels through which such engagement currently operates.

Third, the study contributes to ongoing theoretical discussions by conceptualizing scientific diasporas as “infrastructural mediators”, actors who not only transfer knowledge across borders but reconfigure the relational conditions under which knowledge circulates and becomes actionable. This perspective moves beyond the enabling narratives that often characterize brain circulation and linkage frameworks by foregrounding the structural asymmetries within which diaspora engagement unfolds and by which it is constrained.

## Data Availability

The raw data supporting the conclusions of this article will be made available by the authors, without undue reservation.
