# Supplementary material for: Scientific diasporas in global biodiversity governance: brain circulation, linkage, and knowledge brokerage in Colombia
Source: Front Res Metr Anal. 2026 Jul 3;11:1844103. doi: 10.3389/frma.2026.1844103 (PMC13375901; doi:10.3389/frma.2026.1844103)
Supplement: Supplementary file 1 [file Table_1.docx]

**Annex 1**

**Typology of Diaspora Collaboration Mechanisms in Biodiversity Research**

| **Collaboration mechanism** | **Description** |
| --- | --- |
| Academic collaboration | Formal institutional partnerships that support joint research and researcher mobility via co‑supervised doctoral candidates, undergraduate mentorship, research stays, courses and symposia, participation in MinCiencias‑recognized research groups, and joint grant applications. Such collaborations are governed by formal agreements (e.g., memoranda of understanding, co‑tutelle arrangements) and seek to generate scholarly outputs, strengthen long‑term research capacity, and create institutional linkages between universities and research centers. |
| Capacity building and technology transfer | Activities that combine human and institutional capacity development with mechanisms to translate research‑derived knowledge into practice, e.g., training workshops, licensing, and joint innovation with industry. The emphasis is on strengthening scientific research and implementation capacities, so that transferred technologies or methods become locally usable, maintainable, and improvable. |
| Co-production with society | Participatory research approaches where scientists and non‑academic actors such as citizens, local communities, NGOs, and indigenous groups jointly define research questions, collect and interpret data, and mobilize findings for local use. This includes citizen‑science documentation, community advocacy support, and co‑created educational materials for local communities. The focus is on socially relevant outcomes and legitimacy through stakeholder engagement, prioritizing shared decision‑making, and mutual learning over unidirectional knowledge transfer. |
| Material request | Requests, transfers, or loans of physical samples, e.g., plant specimens, microbial strains, soil, water, and associated data. These transactions typically require permits, material transfer agreements, and documentation of provenance. |
| Policy advice | Activities in which researchers synthesize evidence and provide targeted inputs to policy processes such as policy briefs, technical reports, expert testimony, participation in advisory panels, or co‑development of policy options. The focus is on translating scientific findings into actionable recommendations and engaging decision‑makers at the right time. |
| Non-institutional collaboration | Informal, individual collaborations that arise from personal networks rather than institutional agreements. Typical examples include informal technical consultations, short researcher visits arranged between colleagues, and mentoring that is not embedded in institutional programs. These ties are often flexible and responsive but harder to sustain over time. |

**Annex 2**

**Policy recommendations**

| **Ministries of Environment** | |
| --- | --- |
| **Recommendation** | **Fosters collaboration mechanism** |
| Creation of a permit scheme for the collection and export of samples specifically designed for the scientific diaspora, with an online application platform | - **Academic collaboration:** reduces delays for joint research projects. - **Non-institutional collaboration:** facilitates peer collaborations. - **Material request:** directly improves ABS, MTAs, and permit management. |
| Establish funds and financing programs for the storage and preservation of biological collections in Colombia under appropriate conditions | - **Academic collaboration**: enables sustained research agendas based on well-maintained collections. - **Capacity building & technology transfer**: strengthens infrastructure for long-term preservation. - **Material request**: enhances national sovereignty over genetic resources. |
| Capitalise on existing international digital platforms for information reporting (e.g., Geo BON), encouraging open, standardised, and rapid sharing of both data and analytical tools | - **Academic collaboration:** improves data availability for joint research. - **Capacity building & technology transfer:** supports data standardization, interoperability, and monitoring; democratizes access to data and tools. - **Co-production with society:** Enhances community empowerment and monitoring capacity; enables scientists to obtain data more efficiently; |
| Establish dedicated policies for the discovery and protection of fungal diversity, recognizing that undocumented biodiversity may still be threatened and may also represent an economic opportunity for territorial development. | - **Academic collaboration:** supports joint research on fungal diversity. - **Capacity building & technology transfer:** strengthens national capacity for fungal discovery and monitoring. - **Co-production with society:** encourages collaboration with local communities and knowledge holders. - **Policy advice:** improves evidence-based protection of understudied groups such as fungi and strengthens national biodiversity governance. |

| **Ministries of Science, Technology, and Innovation (STI)** | |
| --- | --- |
| **Recommendation** | **Fosters collaboration mechanism** |
| Establishment of a roadmap for responsible bioprospecting that integrates conservation, innovation, and industrial development objectives, guiding researchers in the transition from biodiversity-based discoveries to marketable products | - **Capacity building & technology transfer :** strengthens national capabilities for translating discoveries into applications. - **Co-production with society:** enables collaboration with local actors in early discovery and benefit-sharing processes. |
| Establishment of a long-term STI policy that goes beyond the priorities of a single administration and balances technological advancement with local needs | - **Academic collaboration:** builds trust among researchers through institutional commitment; generates context-relevant impact. - **Co-production with society:** aligns technological development with and community priorities. - **Policy advice**: strengthens alignment between research and national development goals; increases policy uptake of scientific results. |
| Creation of long-term funding programs, including opportunities for partnerships with civil society, and co-financing with the private sector | - **Academic collaboration**: supports sustained joint research; reduces asymmetries in collaborations. - **Capacity building & technology transfer:** enables infrastructure investment, and innovation. - **Co-production with society:** accelerates discovery; highlights the value of commercially unexplored biological resources. |
| Promote and finance annual meeting spaces for the scientific diaspora together with institutions in Colombia, focused on prioritized thematic areas | - **Non-institutional collaboration:** strengthens peer networks and builds trust. - **Policy advice:** creates structured channels for expert input; catalyzes standard-setting dialogues about systemic barriers. |
| Create mechanisms that allow the scientific diaspora to contribute to strengthening the National STI System. | - **Academic collaboration:** supports agenda-setting and project development. - **Capacity building & technology transfer:** supports multi-directional knowledge flow. - **Policy advice:** integrates diaspora expertise into national governance. |
| Support the organization and science diplomacy training of the scientific diaspora to participate as accredited stakeholders or observer organizations in global science–policy platforms such as IPBES. | - **Academic collaboration:** enhances the diaspora’s role in co-producing assessments, synthesizing evidence, and shaping research agendas. - **Capacity building & technology transfer**: builds technical and procedural skills accelerating knowledge transfer back to Colombian institutions. - **Policy advice**: increases Colombia’s visibility and influence in international biodiversity governance. |
| Promote the development of digital decision-support infrastructures, including simulation platforms and cognitive digital twins, to integrate biodiversity data, socio-economic variables, and policy scenarios. | - **Academic collaboration**: enables integration of interdisciplinary research into applied decision-making tools. - **Capacity building & technology transfer**: strengthens national capabilities in data science, modelling, and simulation. - **Co-production with society**: facilitates participatory scenario analysis involving local communities and stakeholders. - **Policy advice**: supports evidence-based policymaking through scenario evaluation and impact assessment. |
